# Supplementary material for: The holocephalan ratfish endoskeleton shares trabecular and areolar mineralization patterns, but not tesserae, with elasmobranchs little skate and catshark
Source: eLife. 2025 Oct 27;13:RP94900. doi: 10.7554/eLife.94900 (PMC12558651; doi:10.7554/eLife.94900)
Supplement: Supplementary file 1. [file elife-94900-supp1.docx]

**Supplemental File 1. Measurements and regions of interest of specimens**

| **Specimens** | **Sample size** | **Total length TL and disc width DW measurements** | **Regions of interest** |
| --- | --- | --- | --- |
| Little skate stage 32 embryos | 5 | 3.2 cm DW, 7.5 cm TL  2.8 cm DW, 7 cm TL  2.8 cm DW, 7.3 cm TL  3 cm DW, 7.3 cm TL  2.9 cm DW, 7.3 cm TL | Caudal vertebrae |
| Little skate stage 33 embryos | 5 | 3.5 cm DW, 8 cm TL  3.6 cm DW, 7.8 cm TL  3.4 cm DW, 7.8 cm TL  3.4 cm DW, 7.5 cm TL  3.3 cm DW, 7.9 cm TL | Caudal vertebrae |
| Little skate juveniles | 5 | 5.5 cm DW, 10.4 cm TL  5.6 cm DW, 10 cm TL  6 cm DW, 10.5 cm TL  6 cm DW, 11 cm TL  6.5 cm DW, 11 cm TL | Caudal vertebrae |
| Little skate adults | 4 | 43.5 cm TL  45 cm TL  47 cm TL  47.5 cm TL | Precaudal vertebrae, caudal vertebrae |
| Small-spotted catshark | 3 | 31 cm TL  31 cm TL  31 cm TL | Ceratohyal, precaudal vertebrae, caudal vertebrae |
| Spotted ratfish | 5 | 28.5 cm TL  32 cm TL  33 cm TL  40 cm TL  45 cm TL | Precaudal vertebrae, pharyngeal skeleton, ceratohyal, and synarcual |
